# AAA Research Methodology II: Innovative Observational and Intervention Studies

**DOI:** 10.1093/geroni/igaa057.2968

**Published:** 2020-12-16

**Authors:** Wenjun Li, Vivian Lou

## Abstract

Asians are the largest and the fastest growing segment of the world population, and the second largest immigrant population in the U.S. A high proportion of Asians live in developing countries or are immigrants to developed countries. Studies on age-related social and health issues of Asians have lagged. To advance research into aging among Asians living in the U.S. and elsewhere in the world, sound methodologies can be adopted from those well-developed in other settings while novel methodologies are to be developed to meet the unique needs of Asian studies. This symposium brings together five abstracts that address a variety of innovative methodological issues in social and health studies among Asian older adults. The topics range from use of wearable devices to measure travel patterns, life space and physical activity in late-life Asian immigrants in the U.S.; use of Ecological Momentary Assessment method to examine influences of social contact satisfaction on neighborhood affect in Chinese in mainland China; use of multilingual age-friendly touch-screen device for cognitive intervention in Singapore; use of GIS to evaluate potential influences of accessibility to neighborhood living resources on attitudes towards aging among the oldest old in Shanghai; and use of a multi-phase complex mixed methods to evaluate a Chinese cancer screening program in Taiwan. Together, these studies showed that innovative methods can be adapted for context relevance and linguistic and cultural appropriateness, and successfully executed in studies on aging among Asians. This symposium is a collaborative effort of the Aging Among Asians Interest Group.

